# Therapeutic Approach of a High Functioning Individual With Traumatic Brain Injury and Subsequent Emotional Volatility With Features of Pathological Laughter and Crying With Dextromethorphan/Quinidine

**DOI:** 10.1097/MD.0000000000002886

**Published:** 2016-03-25

**Authors:** Dynela Garcia-Baran, Thomas M. Johnson, Joyce Wagner, Joann Shen, Michelle Geers

**Affiliations:** From the Regimental Aid Station, NHCL (DG-B); Department of Neurology, NHCL Intrepid Spine Concussion Recovery Center (TMJ, JW); and Speech-language Pathologist (JS, MG).

## Abstract

Pathological laughing and crying, or pseudobulbar affect (PBA), has been described in patients with neurological disorders such as multiple sclerosis, amyotrophic lateral sclerosis, Alzheimer's disease, stroke, and traumatic brain injury (TBI) since the 19th century (Schiffer 2005). The syndrome is characterized by inappropriate episodes of laughing or crying after minor stimuli. It was first coined a disinhibition of cortical control by Kinnier Wilson in 1924. It was observed in brain disease and seen with mild TBI. It can impair social and occupational function and is largely underrecognized in clinical settings. PBA is usually treated with antidepressants and dopaminergic agents. In this case we treated a military recruit with TBI with Nuedexta—a dextromethorphan/Quinidine derivative with a subsequent decrease in his episodes.

## INTRODUCTION

Pathological laughing and crying, or pseudobulbar affect (PBA), has been described in patients with a variety of neurological disorders, including multiple sclerosis (MS), amyotrophic lateral sclerosis (ALS), epilepsy, brain tumors, Alzheimer's disease, and traumatic brain injury (TBI) since the 19th century. PBA is characterized by uncontrollable, inappropriate episodes of laughing or crying after minor stimuli that would not typically cause such an outburst and are embarrassing or socially awkward when they occur for the individual with PBA. Dextromethorphan/Quinidine (DM/Q) has been demonstrated to be effective in the treatment of PBA in individuals with ALS or MS.^1^ We report a case of an individual who was assaulted and suffered a TBI and subsequently developed PBA who was successfully treated with DM/Q.

## CASE PRESENTATION

A 34-year-old male Marine presented to the Intrepid Spirit Concussion Recovery Center, a holistic, integrated, interdisciplinary clinic, for treatment in April 2012 following an assault in November 2011. He reported having little memory of events for about 2 days before the assault to about 2 days after the assault. History obtained from the chart documented that he had been struck in the head and suffered a traumatically induced physiological disruption of brain function, thereby fulfilling the diagnostic criteria of TBI.

He complained of headaches, disturbances in sleep, increased irritability, difficulty with short-term memory, and occasional episodes of dizziness that all started after his assault. He also stated that before his TBI he could take unpleasant feelings and memories of frightening, stressful situations, and metaphorically “place them in a filing cabinet, stick them in there, shut the door” in his mind and proceed with his daily activities with no problems. However, after the TBI he could not “shut the door anymore” and began experiencing enormous emotional volatility.

In addition, after the assault he began having episodes of crying at unusual or inappropriate times and episodes of laughter after minor stimuli that were embarrassing to him. His past medical history was remarkable for the diagnosis of posttraumatic stress disorder (PTSD) which started after the assault, as well as various orthopedic injuries, including an ankle injury. He was taking prazosin 2 mg qhs, nortriptyline 50 mg qhs, and eszopiclone 2 mg qhs when he presented to the clinic. He had no known drug allergies.

**FIGURE 1:**
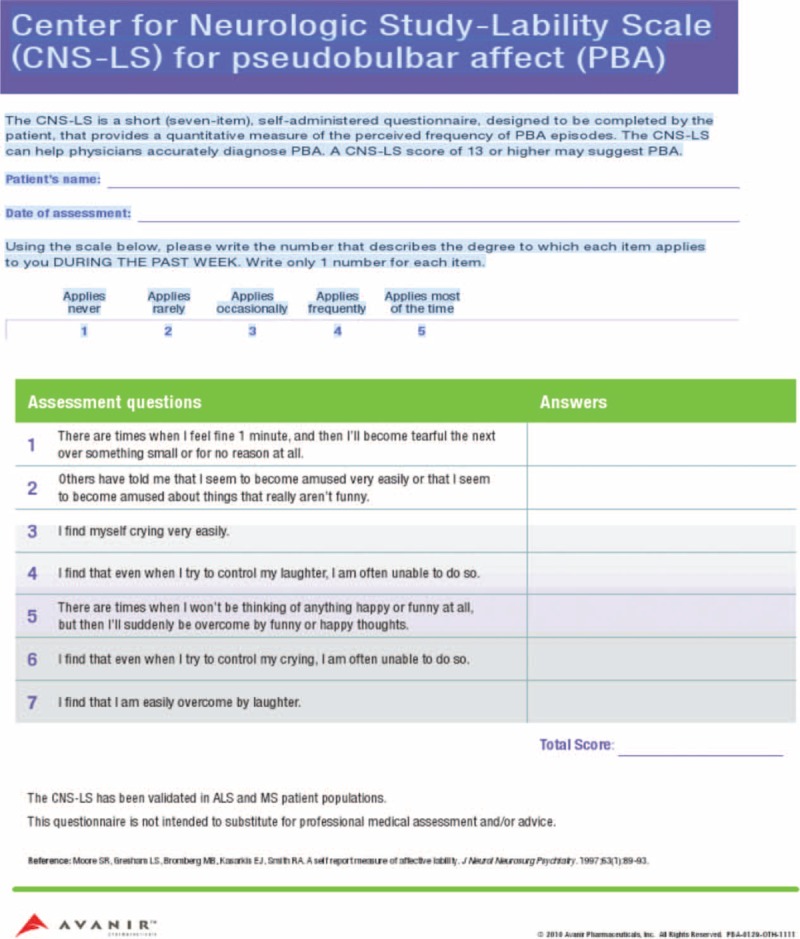
CNS-LS for PBA. CNS-LS = Center for Neurologic Study-Lability Scale, PBA = pseudobulbar affect.

**FIGURE 2:**
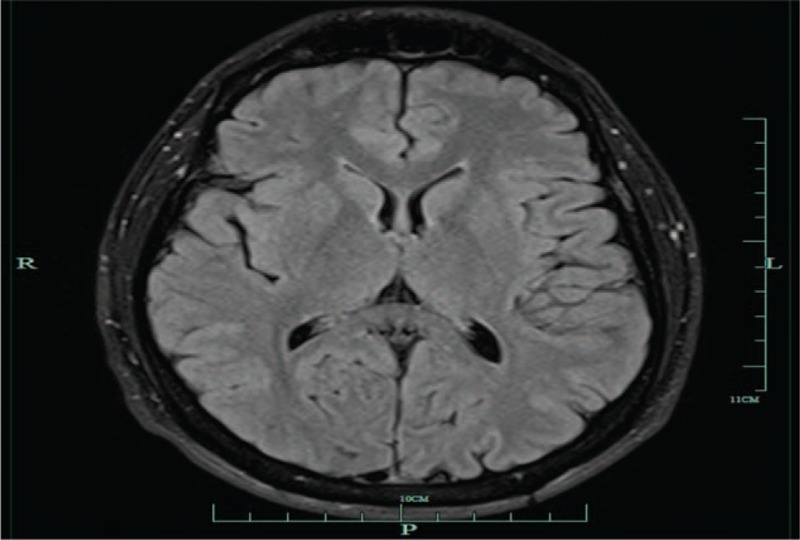
Small lesion in the right frontal deep white matter on the T2 and Flair images which measure 2 to 3 mm in size. Ten percent normal individuals under the age of 40 have a similar foci (TK Burkhard, MD, unpublished data, March 2012).

Physical examination was remarkable only for antalgic gait due to an orthopedic injury to his ankle. His neurological examination was remarkable for the Center for Neurologic Study-Lability Scale (CNS-LS) for PBA of 26, where CNS-LS of 13 or higher is highly suggestive of PBA (Figure 1). His funduscopic examination, cranial nerve, motor, sensory, and cerebellar examination were all normal. He had some difficulty with Romberg testing and gait because of his ankle injury. His reflexes were 2/4, and Babinski testing demonstrated downgoing toes. Laboratory testing was unremarkable, and electrocardiogram was normal. Magnetic resonance imaging of the brain revealed an area of increased signal in the right frontal lobe. However, these lesions had described previously as age appropriate. They have also been seen and described in PBA (Figure 2).

He started taking 20 mg DM/10 mg Q, 1 tablet a day. He felt better after taking this medication for several weeks so he discontinued the medication on his own accord, but then the symptoms of PBA returned. He again started taking 20 mg DM/10 mg Q and was titrated up to twice a day dosing. He is currently on this dose of the medication, and reports that he is able to “shut the door” again and control his emotional volatility when taking the medication, and that the symptoms of PBA have resolved. He reported no side effects or problems with taking the medication.

## DISCUSSION

The regulation of mood and affect is a complicated and poorly understood phenomenon. It probably involves at the very least emotional input from the limbic system that is projected to the frontal cortex; projections from the frontal cortex via the corticobulbar tracts to the cranial nerves in the brainstem resulting in facial nerve activation manifesting as laughing or crying, with additional input from the cerebellum modulating motor activity.

The exact neuroanatomy involved in the regulation of mood and affect is unclear, but there is a consensus in the literature that it involves several areas of the brain functioning as a neural network with multiple feedback loops regulating cortical activity. Such a network would be vulnerable to coup-countercoup injury, as well as axonal shear injuries, which is commonly seen in TBI.

The neuropharmacology of all the receptors and neurotransmitters involved in the regulation of mood and affect is probably even less understood than the neuroanatomy. However, there is evidence that tonic activation of the N-methyl-d-aspartate glutamate (NMDA) receptor can cause neuronal injury and death.^2^ Phasic activation of the receptor can cause learning difficulties and learning impairment.

DM is a known low affinity noncompetitive antagonist of the NMDA receptors. It is also a sigma receptor agonist, and has been shown to have a neuroprotective effect on injured brain.^3^ Q is a potent cytochrome P450 2 D6 inhibitor, and slows the metabolism of DM, resulting in higher levels of DM for longer periods in the bloodstream; 20 mg of DM and 10 mg of Q were married to create DM/Q (Nuedexta).

The most common side effect of DM/Q is dizziness, nausea, and headaches. The most concerning side effect is from Q, which has been used to treat atrial fibrillation and flutter, but can cause prolonged QT syndrome in the 1000 mg/d dosage range. However, the dose of Q in DM/Q is approximately 100 times less than the order of magnitude typically associated with cardiac side effects.

Based on this pharmacological information, DM/Q was evaluated and found in 3 different double blind, randomized clinical studies to significantly improve the symptoms of PBA in people suffering from MS or ALS. There is a paucity of data about the use of DM/Q in TBI, especially in high functioning individuals with TBI such as the individual described in this case report.^1,4,5^

## CONCLUSION

This Marine had a TBI with complete amnesia of events surrounding and including the assault that caused the TBI. He then presented with classic symptoms seen after a TBI, including headaches, problems with mood, memory, and sleep. He also gave a remarkably insightful description of intrusive thoughts and memories that he could longer “close the door” on and control that is consistent with his diagnosis of PTSD.

Such scenarios, where an individual does not consciously recall a trauma that they have experienced and then go on to develop PTSD, are not uncommon, and may be due to the individual striving to cope with the repercussions of the trauma. This individual had an improvement in not just his PBA, but also ability to manage intrusive memories, when taking DM/Q. This suggests that individuals who have developed PBA as a result of TBI could potentially benefit from DM/Q. It also raises the possibility that there is a neuroanatomical and/or neurophysiological disorder that manifests itself clinically as PTSD that responds to DM/Q.

There is no evidence that this individual experienced any adverse effects or harm from taking DM/Q. He was being treated with several medications and receiving cognitive, occupational, vestibular, and other therapies at the same time he was treated with DM/Q, so it is not possible to attribute his clinical improvement solely to the DM/Q. However, the fact that he discontinued the medication and then had a worsening of symptoms, and then restarted the medication with improvement of symptoms, suggests that he was benefiting from the DM/Q. Further studies are required to determine if DM/Q is effective in the treatment of PBA in TBI patients.
